# Intravenous pulse methylprednisolone for induction of remission in severe ANCA associated Vasculitis: a multi-center retrospective cohort study

**DOI:** 10.1186/s12882-019-1226-0

**Published:** 2019-02-18

**Authors:** Dimitrios Chanouzas, Julie Anne G. McGregor, Peter Nightingale, Alan D. Salama, Wladimir M. Szpirt, Neil Basu, Matthew David Morgan, Caroline J. Poulton, Juliana Bordignon Draibe, Elizabeth Krarup, Paula Dospinescu, Jessica Anne Dale, William Franklin Pendergraft, Keegan Lee, Martin Egfjord, Susan L. Hogan, Lorraine Harper

**Affiliations:** 1 Institute of Clinical Sciences, University of Birmingham, Birmingham, UK; 2 University Hospitals Birmingham NHS Foundation Trust, Birmingham, UK; 3 University of North Carolina Kidney Center, Chapel Hill, North Carolina USA; 4 Centre for Nephrology, University College London, London, UK; 5 Copenhagen University Hospital, Copenhagen, Denmark; 6 Institute of Infection, Immunity and Inflammation, University of Glasgow, Glasgow, UK

**Keywords:** ANCA, Vasculitis, Methylprednisolone, Infection, Diabetes mellitus

## Abstract

**Background:**

Intravenous pulse methylprednisolone (MP) is commonly included in the management of severe ANCA associated vasculitis (AAV) despite limited evidence of benefit. We aimed to evaluate outcomes in patients who had, or had not received MP, along with standard therapy for remission induction in severe AAV.

**Methods:**

We retrospectively studied 114 consecutive patients from five centres in Europe and the United States with a new diagnosis of severe AAV (creatinine > 500 μmol/L or dialysis dependency) and that received standard therapy (plasma exchange, cyclophosphamide and high-dose oral corticosteroids) for remission induction with or without pulse MP between 2000 and 2013. We evaluated survival, renal recovery, relapses, and adverse events over the first 12 months.

**Results:**

Fifty-two patients received pulse MP in addition to standard therapy compared to 62 patients that did not. There was no difference in survival, renal recovery or relapses. Treatment with MP associated with higher risk of infection during the first 3 months (hazard ratio (HR) 2.7, 95%CI [1.4–5.3], *p* = 0.004) and higher incidence of diabetes (HR 6.33 [1.94–20.63], *p* = 0.002), after adjustment for confounding factors.

**Conclusions:**

The results of this study suggest that addition of pulse intravenous MP to standard therapy for remission induction in severe AAV may not confer clinical benefit and may be associated with more episodes of infection and higher incidence of diabetes.

**Electronic supplementary material:**

The online version of this article (10.1186/s12882-019-1226-0) contains supplementary material, which is available to authorized users.

## Background

The antineutrophil cytoplasmic antibody (ANCA) associated vasculitides (AAV) are a group of systemic, autoimmune, inflammatory conditions, that include granulomatosis with polyangiitis (GPA), microscopic polyangiitis (MPA) and its renal limited variant (renal limited vasculitis; RLV). They are characterised by necrotising inflammation affecting small to medium blood vessels leading to end organ damage [1].

Severity in AAV ranges from localised disease to severe involvement, the latter defined by the presence of life threatening manifestations that usually include rapidly progressive glomerulonephritis with a requirement for renal replacement therapy. Treatment in AAV is dictated by severity [2]. The MEPEX trial compared plasma exchange (PEX) to intravenous pulse methylprednisolone (MP) over 3 days in addition to oral cyclophosphamide and high dose oral corticosteroids for induction of remission in severe AAV and showed improved renal recovery in the PEX arm [3]. Since then, the use of PEX for induction of remission in severe AAV has become commonplace, albeit on a recent follow-up analysis the long-term benefit of PEX has been less clear [4]. However, many clinicians also routinely use MP prior to commencing high dose oral corticosteroids in addition to PEX and cyclophosphamide.

Use of high dose corticosteroids, including MP, must be balanced with their extensive side effect profile and significant resultant toxicity. Several studies in AAV have suggested that corticosteroids are directly linked to an increased risk of infection [5–9] whilst weight gain, hyperglycaemia and new onset diabetes mellitus represent important metabolic adverse effects of corticosteroids with significant consequences for patients [5, 9–11]. Furthermore, infection remains the most common cause of death in the first 12 months following diagnosis of AAV [12, 13].

A single-centre analysis from 1989 of a heterogeneous cohort of patients with acute crescentic rapidly progressive glomerulonephritis of various aetiologies suggested better outcome in patients treated with MP [14]. However, since then there have been no randomised controlled trials or observational studies in AAV patients examining whether the use of MP confers benefit for patients, or indeed whether it is associated with harm.

Given the paucity of information in the literature, we sought to determine whether the addition of intravenous pulse MP to standard therapy for induction of remission in severe AAV is associated with an improvement in survival, renal recovery or relapse within the first year after diagnosis and examine any possible association with treatment related adverse events such as infection, leukopenia and new onset diabetes mellitus.

## Methods

### Patients

We retrospectively analysed outcomes of consecutive patients that presented in five renal centres in Europe and the United States with a new diagnosis of severe AAV and that received standard therapy for induction of remission with or without intravenous pulse MP between 2000 and 2013.

Definition of severe AAV was based on clinical features in keeping with a diagnosis of AAV (granulomatosis with polyangiitis, microscopic polyangiitis or renal limited vasculitis) and either positive ANCA serology or characteristic features on biopsy, as well as a creatinine greater than 500 μmol/L or dialysis dependency at presentation. Patients were classified based on ANCA specificity (PR3 or MPO) rather than clinical diagnosis as accumulating evidence suggests that ANCA specificity affects the phenotype of clinical disease, as well as the patient’s initial response to remission-induction therapy and relapse risk [15]. Patients had to have received PEX, pulse or continuous oral cyclophosphamide and high-dose oral corticosteroids with or without intravenous pulse MP for inclusion in the study. We excluded patients that received rituximab or a combination of rituximab and cyclophosphamide for induction of remission. Intravenous pulse MP use was dependent on physician choice. Use of intravenous pulse MP across the 5 study centres is shown in Additional file 1: Table S1.

Data was collected retrospectively from electronic and paper medical records. Birmingham Vasculitis Activity Score (BVAS) was calculated retrospectively. We collected data on baseline descriptive and treatment characteristics as well as pre-defined outcomes over the first 12 months post AAV diagnosis and commencement of therapy as follows: survival, renal recovery, relapses, infections, leukopenic episodes (white cell count < 4 × 10^9^/L) and diagnosis of new onset diabetes mellitus.

Renal recovery at 12 months was defined as achievement of independence from renal replacement therapy and no further requirement for renal replacement therapy by 12 months for patients that required dialysis at presentation, or, improvement in renal function and no requirement for renal replacement therapy by 12 months for patients that presented with creatinine greater than 500 μmol/L but were not dialysis dependent at presentation. We also collected data on duration of dialysis for patients that were dialysis dependent at presentation, and creatinine at 12 months. When examining creatinine at 12 months we excluded patients that were on dialysis. Retrospective clinical information regarding episodes of infection was obtained by examining hospital medical records. All significant documented infections (requiring antibiotic treatment or admission to hospital) were included. Severe infections were defined as those requiring hospital admission, prolongation of hospital admission or treatment with intravenous antibiotics. We defined new onset diabetes as a requirement for treatment with either oral hypoglycaemic agents or insulin in a patient not diagnosed with diabetes mellitus prior to commencing induction treatment for AAV.

### Data presentation

Data was analysed using SPSS Version 21 (Armonk, NY: IBM Corp.), GraphPad Prism Version 5 (GraphPad Software, San Diego, California) and R (R Foundation for Statistical Computing, Vienna, Austria). Baseline characteristics, renal recovery and relapses between MP treated and non-MP treated patients were compared using the Chi-square test, or Fisher’s exact test where appropriate, for categorical variables, and the Mann Whitney U test for non-parametric continuous variables. Kaplan-Meier curve analysis was employed to examine differences in survival, time to infection, severe infection, leukopenia and new onset diabetes in the two groups. Patients with pre-existing diabetes were excluded from the analysis for new onset diabetes. Log rank was used for statistical comparisons of Kaplan-Meier curves.

Proportional hazards analysis was used to detect significant predictors of time to first infection, severe infection, new onset diabetes and survival. Time dependent covariates were entered in models in a time dependent fashion. Cyclophosphamide dose and oral prednisolone dose were entered as segmented time dependent covariates as follows: cyclophosphamide dose at 2 weeks, 1 month, 3 months and 6 months and oral prednisolone dose at 1 week, 2 weeks, 1 month, 3 months, 6 months and 12 months. Multivariable proportional hazards models were constructed to control for relevant confounders. Covariates with a *p* value ≤0.1 on univariable analysis were included in the models. A p value of less than 0.05 was considered statistically significant for all analyses.

## Results

### Baseline and treatment characteristics

Baseline characteristics for 114 patients that presented with severe AAV and fulfilled the inclusion criteria for the study are shown in Table 1. Fifty-two patients received intravenous pulse MP in addition to standard therapy for induction of remission compared to 62 patients that did not. There were no differences in age or gender between groups but there was a statistically significant difference in ethnicity. Clinical characteristics and markers of severity such as creatinine at baseline, percentage of glomeruli with crescents on initial renal biopsy, and BVAS score were comparable except for patients that received MP were more likely to have had concomitant pulmonary haemorrhage but less likely to have had other lung or ENT involvement (Table 1).

**Table 1:** Baseline and treatment characteristics

	Overall	Methylprednisolone treated	No methylprednisolone	*P* value*
Number of patients	114	52	62	
Age, years	62 (54–72)	60 (47–72)	62 (56–72)	0.166
No. of men/women	77/37	35/17	42/20	0.961
No. of Caucasian/Non-Caucasian	100/14	39/13	61/1	< 0.001
No. of PR3 positive (%)	65 (57.0)	27 (51.9)	38 (61.3)	0.314
No. with pulmonary haemorrhage (%)	26 (22.8)	18 (34.6)	8 (12.9)	0.006
No. with other lung disease (%)	59 (51.8)	20 (38.5)	39 (62.9)	0.009
No. with ENT disease (%)	49 (43.0)	15 (28.8)	34 (54.8)	0.005
BVAS score	18 (12–20)	16 (12–22)	20 (14–20)	0.852
Glomeruli with crescents (%)	60.0 (34.5–80)	62.5 (33.0–77.0)	58.5 (37.5–80.0)	0.666
Baseline Creatinine, μmol/L	619 (530–790)	594 (506–787)	647 (547–790)	0.189
Plasma Exchange, No. of treatments	7 (6–9)	7 (5–7)	7 (7–10)	< 0.001†
MP dose, g	–	1.5 (1.5–1.9)	–	–
Total Cyclophosphamide dose, g	7.2 (4.6–9.8)	5.2 (3.6–9.4)	8.6 (6.4–10.5)	0.005
Total Oral Prednisolone dose (12 months), g	5.5 (3.9–6.8)	4.0 (3.9–5.3)	6.8 (5.5–7.0)	< 0.001

Values reported as median (IQR)

*Comparison between MP and non-MP treated patients

† Median value was the same in MP and non-MP treated patients but in MP group 3 values were greater than the median compared to 29 values greater than the median in the non-MP group

The median dose of MP was 1.5 g (IQR: 1.5–1.9) administered in 3 intravenous pulses over 3 days. Patients treated with MP received a lower number of PEX cycles compared to non-MP patients and a lower dose of total cyclophosphamide and total oral prednisolone dose over the first 12 months following diagnosis (Table 1). Cumulative dose of cyclophosphamide and oral prednisolone at different time-points during the study is shown in Additional file 1: Table S2.

### Survival, renal recovery and relapses

**Fig. 1:**
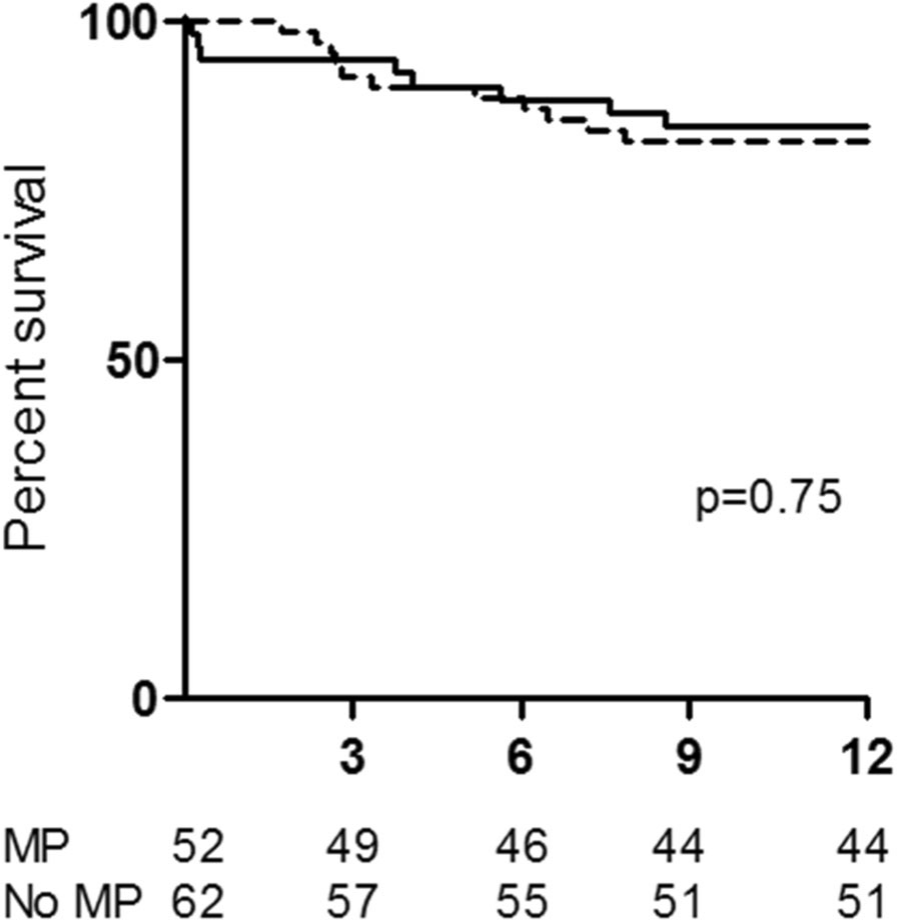
Time to survival by 12 months was examined by Kaplan-Meier curve analysis. Survival for patients that received MP is shown in the dashed line and that for non-MP treated patients in the solid line

There was no difference in overall survival between patients treated with MP and patients that did not receive MP (Fig. 1). At the 3 month time point 94.2% of MP treated patients and 91.9% of non-MP treated patients were alive (*p* = 0.726), whilst at the end of the 12 month follow up those figures were 84.6 and 82.3% respectively (*p* = 0.737). Similarly, there was no difference in renal recovery with 57.7% of MP treated patients and 66.1% of non-MP treated patients having achieved renal recovery by 12 months (*p* = 0.355), although creatinine at 12 months (excluding patients on dialysis) was higher in the MP treated group (MP: 207 μmol/L, IQR 142–269; non-MP: 143 μmol/L, IQR 118–179; *p* = 0.008). In addition, there was no difference in time to renal replacement therapy independence amongst dialysis dependent patients at diagnosis, between MP and non-MP treated patients (MP: 30 days, IQR 14–60; non-MP: 30 days, IQR 12–60; *p* = 1.00). Relapses were similar in the two groups with 11.5 and 8.1% of MP and non-MP treated patients having relapsed by 12 months respectively (*p* = 0.545).

### Adverse events

#### Infection and leukopenia

**Fig. 2:**
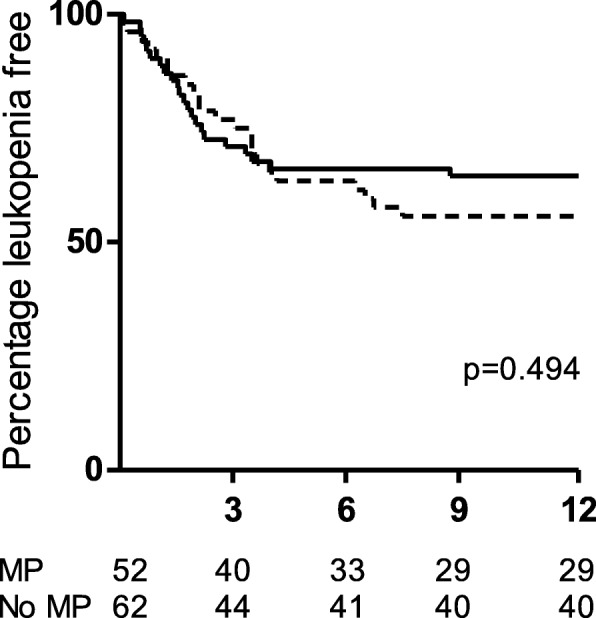
Time to leukopenia by 12 months was examined by Kaplan-Meier curve analysis. Time to leukopenia for patients that received MP is shown in the dashed line and that for non-MP treated patients in the solid line

**Fig. 3:**
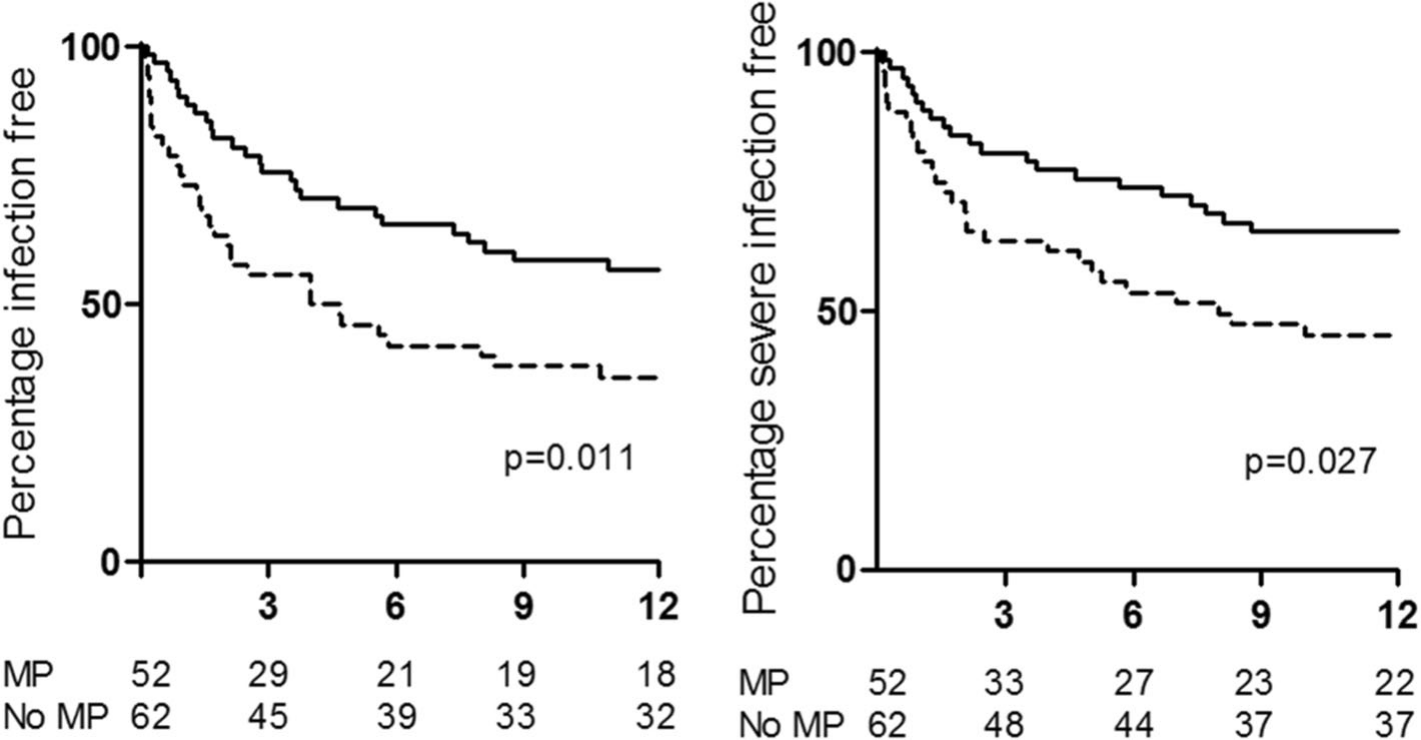
Time to infection overall (left panel) and severe infection (right panel) by 12 months was examined by Kaplan-Meier curve analysis. Time to infection is shown in the dashed line for patients that received MP and in the solid line for non-MP treated patients. The difference in infection is confined to the first 3 months as can be seen by the curves being essentially parallel from 3 months onwards

There was no difference in episodes of leukopenia between MP and non-MP treated patients (Fig. 2). Treatment with MP however was associated with more episodes of infection overall as well as more severe infections. This difference was confined to the first 3 months following diagnosis and commencement of therapy as can be seen in Fig. 3, with 44.2 and 24.2% of MP and non-MP treated patients respectively having had an episode of infection by 3 months. Similarly, 36.5% of MP treated patients compared to 19.4% of non-MP treated patients had an episode of severe infection by the end of 3 months (Fig. 3). There was no difference in the onset of new infections or that of new severe infections between 3 and 12 months (Additional file 1: Figures S1B and S1D). Therefore, multivariable proportional hazards models were constructed to determine independent predictors of infection and severe infection by 3 months.

Variables associated with infection and severe infection by 3 months on univariable analysis are listed in Table 2. When these factors were added in multivariable proportional hazards models, MP treatment remained a significant predictor of infection overall as well as severe infection at 3 months (Table 2). Furthermore the dose of MP delivered was also a significant and independent predictor of infection (hazard ratio (HR) 1.5, 95% CI 1.1–1.9 per gram of MP; *p* = 0.010) and severe infection (HR 1.44, 95% CI 1.03–2.00 per gram of MP; *p* = 0.034) at 3 months (Additional file 1: Table S3).

**Table 2:** Factors associated with infection at 3 months on univariable and multivariable proportional hazards analysis

	Univariable		Multivariable	
	HR (95% CI)	*p* value	HR (95% CI)	*p* value
*(A) Time to infection at 3 months*				
Treatment with MP	2.2 (1.1–4.2)	0.018	2.7 (1.4–5.3)	0.004
Leukopenia preceding infection	3.6 (1.6–8.0)	0.001	4.3 (1.9–9.5)	< 0.001
Dose of oral prednisolone, g	5.4 (1.1–27.3)	0.043	8.5 (1.4–53.2)	0.022
*(B) Time to severe infection at 3 months*				
Treatment with MP	2.1 (1.0–4.3)	0.043	2.9 (1.3–6.6)	0.010
Leukopenia preceding infection	4.8 (2.1–10.9)	< 0.001	5.6 (2.4–13.0)	< 0.001
Dose of oral prednisolone, g	9.2 (1.5–54.7)	0.015	17.9 (1.9–164.5)	0.011
BVAS score	1.1 (1.0–1.2)	0.005	1.1 (1.0–1.2)	0.131
Lung involvement	1.9 (0.9–3.9)	0.096	1.2 (0.5–3.1)	0.678

Dose of oral prednisolone was entered as a segmented time dependent variable as follows: dose at 1 week, 2 weeks, 1 month and 3 months

HR = hazard ratio, CI = confidence intervals

Finally, infection had a significant bearing on survival. Patients that had an infection during the first 3 months had a hazard ratio of 5.58 (95% CI 1.99–16.65; *p* = 0.001) for death by 12 months after adjusting for possible confounders (Table 3).

**Table 3:** Multivariable proportional hazards model for survival at 12 months

	Hazard ratio (95% Confidence Interval)	*P* value
Age, years	1.07 (1.03–1.12)	0.001
Infection by 3 months	5.58 (1.99–15.65)	0.001
Lung involvement	3.53 (1.21–10.31)	0.021
Cyclophosphamide dose, g	0.93 (0.69–1.26)	0.648
Treatment with MP	0.75 (0.28–2.01)	0.564

Dose of cyclophosphamide was entered in the model as a segmented time dependent covariate as follows: dose at 2 weeks, 1 month, 3 months, 6 months and 12 months

Infection during the first 3 months was added as a time dependent covariate indicating whether such an infection had occurred

#### New onset diabetes

**Fig. 4:**
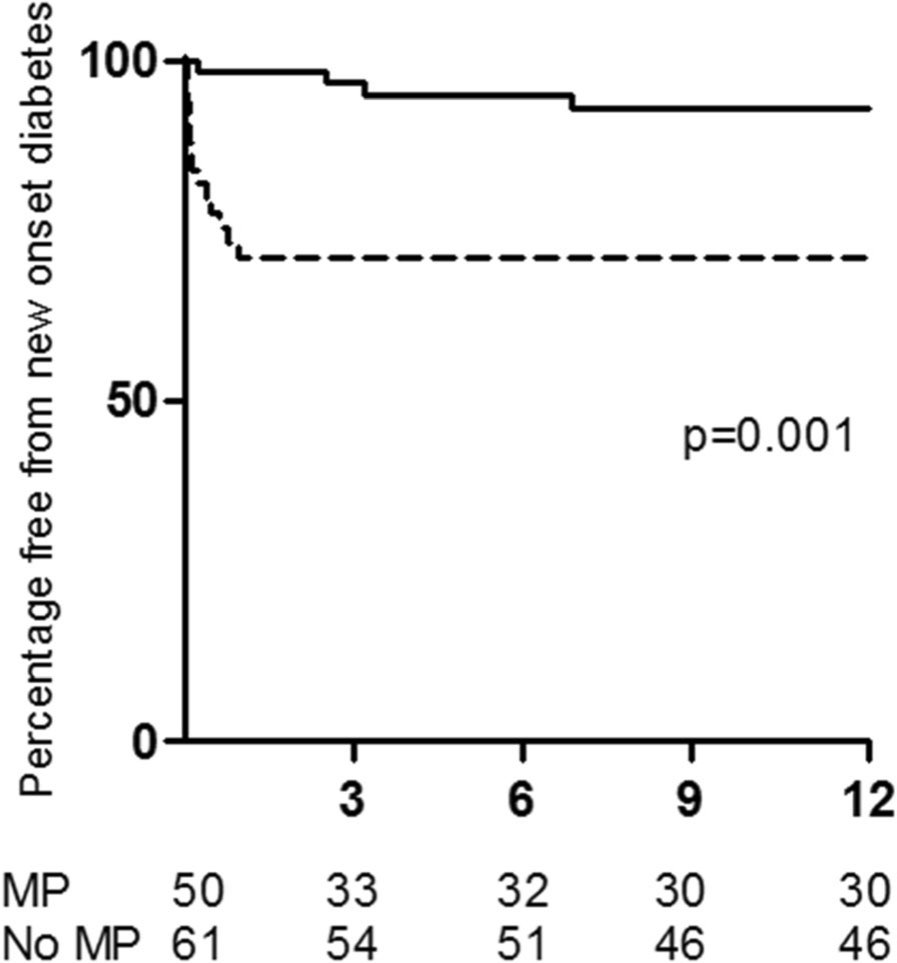
Time to new onset diabetes by 12 months was examined by Kaplan-Meier curve analysis and is shown in the dashed line for patients that received MP and in the solid line for patients that were not treated with MP

We observed an increased incidence of diabetes mellitus amongst MP treated patients. By 12 months 26.9% of MP treated patients developed diabetes compared to 6.5% of non-MP treated patients with most cases occurring within the first month following diagnosis and commencement of therapy as shown in Fig. 4. Treatment with MP was the only factor that was significantly associated with new onset diabetes (HR 5.1 [1.7–15.7], *p* = 0.004). Furthermore, the dose of MP delivered was also a significant predictor of new onset diabetes (HR 1.8, 95% CI 1.2–2.7 per gram of MP; *p* = 0.003). To account for potential confounding factors we also controlled for the effect of age, ethnicity and cumulative dose of oral prednisolone in multivariable proportional hazards models. The association between MP use and MP dose with increased incidence of diabetes mellitus remained significant (Table 4).

**Table 4:** Multivariable proportional hazards model for incidence of diabetes mellitus

	Hazard ratio (95% Confidence Interval)	*P* value
*(A) Treatment with MP*		
Age, years	0.99 (0.96–1.02)	0.621
Ethnicity	1.55 (0.42–5.70)	0.512
Dose of oral prednisolone, g	2.26 (0.47–10.86)	0.310
Treatment with MP	6.33 (1.94–20.63)	0.002
*(B) Dose of MP*		
Age, years	0.99 (0.96–1.02)	0.587
Ethnicity	1.37 (0.37–5.08)	0.640
Dose or oral prednisolone, g	1.85 (0.41–8.44)	0.640
Dose of MP, g	1.90 (1.25–2.90)	0.003

Dose of oral prednisolone was entered as a segmented time dependent variable as follows: dose at 1 week, 2 weeks, 1 month, 3 months, 6 months and 12 months

Ethnicity was classified as Caucasian or non-Caucasian

## Discussion

This retrospective analysis of a large cohort of incident patients who presented with severe AAV in five large vasculitis centres in Europe and the United States suggests that the addition of intravenous pulse MP to standard induction of remission therapy with cyclophosphamide, plasma exchange and high-dose oral corticosteroids may not confer any benefit in terms of improving patient outcomes and may increase patient harm.

We found no difference in overall survival, renal recovery or relapses by 12 months between patients that received MP in addition to standard therapy and those that did not receive MP, despite the two groups being well balanced in terms of age, ANCA specificity and markers of severity such as BVAS score, presenting creatinine and percentage of glomeruli with crescents on renal biopsy. In addition, treatment with MP, as well as the total dose of MP delivered, was associated with a higher and earlier onset of infection and severe infection episodes, and a higher incidence of diabetes mellitus in the MP treated patients that were particularly prominent in the first month following commencement of therapy.

There were significant differences between the MP treated patients and those that did not receive MP. In addition, the proportion of patients that received MP differed amongst the study centres. We carefully controlled for baseline and treatment characteristics in our multivariable analyses. However, it is possible that between-centre differences remain that may not have been fully controlled for by our analyses. This together with the retrospective nature of data collection represents one of the main limitations of this study.

MP treated patients were more likely to have had pulmonary haemorrhage at presentation which suggests a bias towards physician choice of therapy amongst patients that are perhaps perceived to have more severe disease, although there was no difference in the severity of renal disease, or overall BVAS score between the two groups.

There were significant differences in the standard treatment that patients received, with MP treated patients receiving a lower load in terms of number of plasma exchange treatments, total dose of cyclophosphamide and total dose of oral prednisolone over the first 12 months likely reflecting differences between treating physicians and treating centres. A greater proportion of non-MP patients received oral daily instead of pulsed cyclophosphamide and this accounted for the higher cumulative cyclophosphamide dose seen in non-MP treated patients. To account for the treatment differences between MP and non-MP treated patients we carefully controlled for cumulative cyclophosphamide and oral prednisolone dose by analysing these variables in a time-dependent manner as segmented time-dependent covariates using the time-intervals tabulated in Additional file 1: Table S2.

Both the use of MP and the cumulative oral prednisolone dose were independent predictors of risk of infection and risk of severe infection by 3 months. Importantly however, the cumulative oral prednisolone dose was comparable between MP and non-MP treated patients up until 3 months (Additional file 1: Table S2) indicating that the effect of MP use on the risk of infection was separate and additional to that associated with the cumulative oral prednisolone dose received. In addition, MP treated patients had a higher incidence of infection as well as diabetes mellitus nearer the time of presentation and MP treatment, suggesting validity of our results. This observation also suggests that the peak of steroid dose early on in the treatment phase may be important in the development of treatment associated complications.

In systemic lupus erythematosus (SLE), a higher cumulative dose of intravenous MP is associated with more infections and the majority of infections occur within the first month following treatment [16]. Systemic parameters present at the time of diagnosis may also compound this effect. It has been shown for example that hypoalbuminaemia can result in increased serum corticosteroid levels [17] possibly explaining the increased risk of complications at treatment onset but also the variability that is often seen between patients.

There is a paucity of information with regards to risk factors for steroid induced diabetes mellitus development in the AAV literature. In transplantation, it is recognised that early hyperglycaemia following MP and oral corticosteroid treatment is associated with increased likelihood of developing new onset diabetes after transplantation [18, 19]. Whilst an early study did not show an association between the total dose or duration of intravenous MP and new onset diabetes after transplantation [20], a more recent study has suggested increased likelihood of new onset diabetes after transplantation with higher MP doses at the time of transplantation [21], which is in agreement with our findings. The response to MP however may vary across individuals depending on other risk factors such as ethnicity [22]. In our cohort, although we observed a statistically significant difference in ethnicity between MP and non-MP treated groups, we did not find any effect of ethnicity on the development of new onset diabetes. However, given the predominance of Caucasian patients in our cohort, the study was not adequately powered to detect differences in new onset diabetes between Caucasians and other ethnic groups.

In contrast to our findings, a recently published retrospective cohort study examining the effect of intravenous pulse MP on renal recovery in Chinese patients presenting with severe AAV, found that the use of MP was associated with a lower percentage of patients remaining dialysis dependent [23]. However, none of the patients in that study received plasma exchange, whilst the patient cohort was comprised of a population with a higher incidence of MPO-ANCA disease and consequently more chronic sclerotic and mixed lesions in renal pathology compared to Western populations [24, 25]. In addition, patients that did not receive pulse MP in the Ma et al. study had smaller kidneys on ultrasound assessment, were less likely to have had a renal biopsy and might have represented cases of late referral with a higher burden of chronic and fibrotic changes, potentially accounting for the inferior renal recovery outcome in non-MP treated patients in that study [23].

The advent of induction of remission treatment strategies based on cyclophosphamide and high-dose corticosteroids has transformed AAV from an almost universally fatal condition to a chronic illness associated with considerable treatment related morbidity. Indeed one of the main goals and challenges in the management of AAV is to minimise treatment related complications and toxicity without compromising efficacy. Corticosteroids remain central in the development of significant toxicity and current clinical trials [26] are investigating whether lower intensity corticosteroid regimes may be equally effective, with an aim of reducing the toxicity burden associated with steroid use. It must be kept in mind that 1.5 g of MP, the commonest total dose of intravenous MP administered over 3 days in the setting of severe AAV, is equivalent to 1.875 g of oral prednisolone or 1 month of high-dose oral prednisolone treatment (60 mg / day).

## Conclusions

The MEPEX trial showed that plasma exchange was more efficacious at 12 months compared with pulse MP in patients with life-threatening renal disease [3]. However, despite no evidence from randomised controlled trials or indeed contemporary observational data to support this, pulse MP is commonly used in addition to plasma exchange. Our retrospective analysis suggests that addition of pulse intravenous MP to standard therapy for remission induction in severe AAV does not confer clinical benefit. Conversely, MP use was associated with more episodes of infection and a higher incidence of diabetes. Our data question the widespread use of pulse MP in the treatment of severe AAV and indicate the need for an appropriately conducted randomised controlled clinical trial to definitively answer the question of whether MP should be used in the treatment of severe AAV and whether there may be a subgroup of patients in whom treatment with MP may be advantageous.

## Additional file

Additional file 1: **Tables S1.** Intravenous pulse methylprednisolone use and dosage. **Table S2.** Cyclophosphamide and oral prednisolone dose in intravenous pulse methylprednisolone (MP) and non-MP treated patients. **Table S3.** Factors associated with infection at 3 months on univariable and multivariable proportional hazards analysis. **Figure S1.** Time to infection by 3 months (A) and from 3 to 12 months (B) and time to severe infection by 3 months (C) and from 3 to 12 months (D) was examined by Kaplan-Meier curve analysis. Time to infection for patients that received MP is shown in the dashed line and that for non-MP treated patients in the solid line. (DOCX 150 kb)

## Abbreviations

AAV: ANCA associated vasculitis
GPA: Granulomatosis with polyangiitis
MP: Methylprednisolone
MPA: Microscopic polyangiitis
PEX: Plasma exchange
RLV: Renal limited vasculitis
SLE: Systemic lupus erythematosus
