# Supplementary material for: Intravenous pulse methylprednisolone for induction of remission in severe ANCA associated Vasculitis: a multi-center retrospective cohort study
Source: BMC Nephrol. 2019 Feb 18;20:58. doi: 10.1186/s12882-019-1226-0 (PMC6378728; doi:10.1186/s12882-019-1226-0)
Supplement: Supplementary file 1 — Tables S1. Intravenous pulse methylprednisolone use and dosage. Table S2. Cyclophosphamide and oral prednisolone dose in intravenous pulse methylprednisolone (MP) and non-MP treated patients. Table S3. Factors associated with infection at 3 months on univariable and multivariable proportional hazards analysis. Figure S1. Time to infection by 3 months (A) and from 3 to 12 months (B) and time to severe infection by 3 months (C) and from 3 to 12 months (D) was examined by Kaplan-Meier curve analysis. Time to infection for patients that received MP is shown in the dashed line and that for non-MP treated patients in the solid line. (DOCX 150 kb) [file 12882_2019_1226_MOESM1_ESM.docx]

**Additional files**

Intravenous Pulse Methylprednisolone For Induction Of Remission In Severe ANCA Associated Vasculitis: A Multi-Center Retrospective Cohort Study

Dimitrios Chanouzas PhD^1,2^, JulieAnne G. McGregor MD^3^, Peter Nightingale PhD^2^, Alan D. Salama PhD^4^, Wladimir M. Szpirt MD^5^, Neil Basu PhD^6^, Matthew David Morgan PhD^1,2^, Caroline J. Poulton MSW^3^, Juliana Bordignon Draibe MD^4^, Elizabeth Krarup MD^5^, Paula Dospinescu MBChB^6^, Jessica Anne Dale MBChB^2^, William Franklin Pendergraft PhD^3^, Keegan Lee MBChB^4^, Martin Egfjord PhD^5^, Susan L. Hogan PhD^3^ and Lorraine Harper PhD^1,2^.

**Additional file Table S1**  Intravenous pulse methylprednisolone use and dosage

|  | MP use | MP dose (g)* |
| --- | --- | --- |
| Birmingham | 9/23 | 1.00 (0.50-1.75) |
| Chapel Hill | 30/32 | 1.50 (1.50-2.19) |
| London | 9/10 | 1.50 (1.50-1.50) |
| Copenhagen | 3/36 | 1.50 (1.50-1.50) |
| Aberdeen | 1/13 | 3.00 (3.00-3.00) |

* Median (IQR) in patients that received intravenous pulse methylprednisolone (MP)

**Additional file Table S2** Cyclophosphamide and oral prednisolone dose in intravenous pulse methylprednisolone (MP) and non-MP treated patients

|  | MP | Non-MP |
| --- | --- | --- |
| Cyclophosphamide (g) |  |  |
| 2 weeks | 1.0 (0.8 – 1.2) | 1.4 (0.8 – 1.5) |
| 1 month | 1.4 (1.0 – 1.8) | 2.7 (1.5 – 3.0) |
| 3 months | 4.5 (3.1 – 4.9) | 6.5 (4.5 – 9.0) |
| 6 months | 5.2 (3.6 – 9.4) | 8.6 (6.4 – 10.5) |
|  |  |  |
| Oral prednisolone (g) |  |  |
| 1 week | 0.4 (0.4 – 0.4) | 0.4 (0.4 – 0.4) |
| 2 weeks | 0.8 (0.8 – 0.8) | 0.8 (0.8 – 0.8) |
| 1 month | 1.8 (1.4 – 1.8) | 1.6 (1.4 – 1.7) |
| 3 months | 3.8 (2.9 – 3.8) | 3.9 (3.0 – 4.1) |
| 6 months | 4.0 (3.9 – 4.0) | 5.6 (4.4 – 6.3) |
| 12 months | 4.0 (3.9 – 5.3) | 6.8 (5.5 – 7.0) |

Median (IQR) shown

Cumulative dose up to the respective time point is shown

**Additional file Table S3** Factors associated with infection at 3 months on univariable and multivariable proportional hazards analysis

|  | Univariable | | Multivariable | |
| --- | --- | --- | --- | --- |
|  | HR (95% CI) | p value | HR (95% CI) | p value |
| *(A) Time to infection at 3 months* |  |  |  |  |
| MP dose (per gram) | 1.4 (1.1 – 1.9) | 0.017 | 1.5 (1.1 – 1.9) | 0.010 |
| Leukopenia preceding infection | 3.6 (1.6 – 8.0) | 0.001 | 3.9 (1.8 – 8.6) | 0.001 |
| Dose of oral prednisolone, g | 5.4 (1.1 – 27.3) | 0.043 | 7.2 (1.2 – 44.7) | 0.035 |
|  |  |  |  |  |
| *(B) Time to severe infection at 3 months* |  |  |  |  |
| MP dose (per gram) | 1.5 (1.1 – 2.1) | 0.016 | 1.44 (1.03 – 2.00) | 0.034 |
| Leukopenia preceding infection | 4.8 (2.1 – 10.9) | < 0.001 | 5.35 (2.34 – 12.23) | < 0.001 |
| Dose of oral prednisolone, g | 9.2 (1.5 – 54.7) | 0.015 | 15.20 (1.71 – 134.99) | 0.015 |
| BVAS score | 1.1 (1.0 – 1.2) | 0.005 | 1.08 (0.97 – 1.20) | 0.169 |
| Lung involvement | 1.9 (0.9 – 3.9) | 0.096 | 0.93 (0.37 – 2.38) | 0.884 |

Dose of oral prednisolone was entered as a segmented time dependent variable as follows: dose at 1 week, 2 weeks, 1 month and 3 months.

HR = hazard ratio, CI = confidence intervals

The differences in infection and severe infection between MP treated patients and patients that did not receive MP were confined to the first 3 months. As shown in Additional file Figure S1, there is a clear difference in infection between the 2 groups at 3 months. There is no difference in the occurrence of new infections from 3 to 12 months between MP and non-MP treated patients.


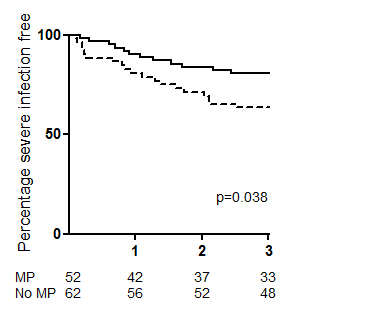

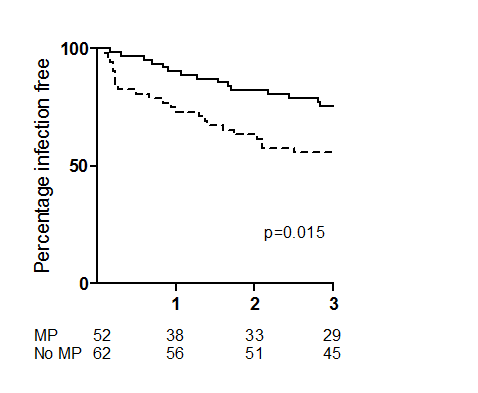

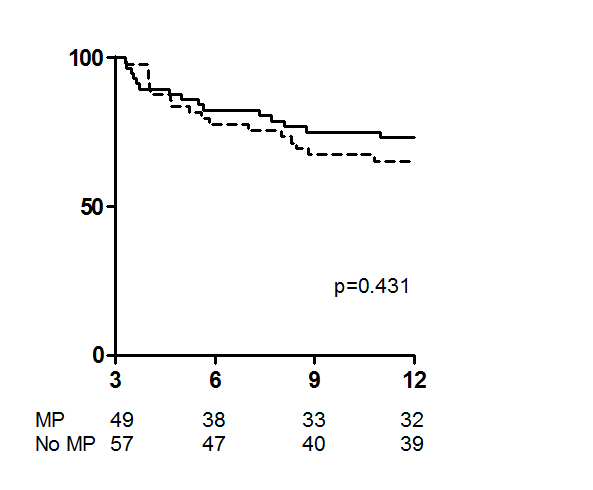

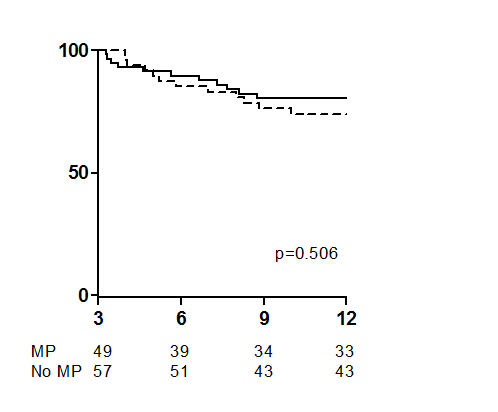


A

B

C

D

**Additional file Figure S1** Time to infection by 3 months (A) and from 3 to 12 months (B) and time to severe infection by 3 months (C) and from 3 to 12 months (D) was examined by Kaplan-Meier curve analysis. Time for infection for patients that received MP is shown in the dashed line and that for non-MP treated patients in the solid line.
